# Octreotide attenuates liver fibrosis by inhibiting hepatic heme oxygenase-1 expression

**DOI:** 10.3892/mmr.2014.2735

**Published:** 2014-10-22

**Authors:** SHI-BIN GUO, QING LI, ZHI-JUN DUAN, QIU-MING WANG, QIN ZHOU, XIAO-YU SUN

**Affiliations:** 1 Department of Gastroenterology, The First Affiliated Hospital of Dalian Medical University, Dalian, Liaoning 0086-116011, P.R. China; 2 Department of Gastroenterology, Dalian Friendship Hospital, Dalian, Liaoning 0086-116011, P.R. China; 3 Department of Pharmacology, Dalian Medical University, Dalian, Liaoning 0086-116011, P.R. China

**Keywords:** heme oxygenase-1, carbon monoxide, octreotide, bile duct ligation, liver fibrosis

## Abstract

The aim of the present study was to investigate the effects of octreotide treatment on hepatic heme oxygenase-1 (HO-1) expression, together with the influence of altered hepatic HO-1 expression levels on hepatic function and fibrosis in bile duct-ligated rats. The rats were divided randomly into sham, cirrhotic, cobalt protoporphyrin and octreotide treatment groups. The expression levels of hepatic HO-1 mRNA were measured by reverse-transcription polymerase chain reaction, while the protein expression was determined by western blotting and immunohistochemical analysis. Hematoxylin and eosin, and Van Gieson’s staining, along with determination of the hydroxyproline content in the liver, were performed to determine the degree of liver fibrosis. The serum levels of alanine aminotransferase (ALT), aspartate aminotransferase (AST), total bilirubin (TBIL) and carboxyhemoglobin (COHb) in arterial blood, and the mean arterial pressure and portal vein pressure were also measured. As compared with the sham group, hepatic HO-1 mRNA and protein expression levels, serum levels of ALT, AST and TBIL, COHb in arterial blood, hydroxyproline and collagen type I content were all significantly increased in the cirrhotic group. As compared with the cirrhotic group, the octreotide-treated group exhibited significantly reduced hepatic HO-1 expression levels, serum levels of ALT, AST and TBIL, COHb in arterial blood and the extent of hepatic fibrosis, whereas the cobalt protoporphyrin group exhibited significantly increased hepatic HO-1 expression levels, as well as aggravated hepatic function and fibrosis (P<0.05). In conclusion, octreotide inhibited hepatic HO-1 overexpression in cirrhotic rats, reduced hepatic HO-1 expression levels to relieve liver injury and attenuated liver fibrosis.

## Introduction

Liver cirrhosis is the final stage of the chronic fibrotic process in the liver and is the predominant cause of portal hypertension. Increased hepatic vascular resistance is the initial factor that establishes portal hypertension and increased portal system blood flow is an important factor in maintaining and aggravating portal hypertension (1). Portal hypertension may result in severe complications, including bleeding from esophageal varices, which is a life-threatening clinical condition and is a major cause of fatality in Asia (2).

Octreotide has been widely used in clinics to control variceal hemorrhage and has been demonstrated to be effective in controlling initial hemorrhage and preventing episodes of re-bleeding (3). Studies have shown that, following either an intravenous bolus administration or a continuous infusion, octreotide significantly reduces portal pressure in patients and experimental animals with portal hypertension (4,5).

Recently, the heme oxygenase/carbon monoxide (HO/CO) system has attracted a great deal of attention. HO-1, which has constitutive and inducible isoforms (6,7), catalyzes the rate-limiting step in the oxidative degradation of heme to biliverdin (8), releasing equimolar quantities of CO and iron (9). Previous studies have shown that hepatic HO-1 was overexpressed in cirrhotic rats (10) and contributed to portal hypertension (11). The present study aimed to investigate whether octreotide administration regulates hepatic HO-1 expression while reducing portal vein pressure (PVP), and the influence of altered hepatic HO-1 expression levels on hepatic function and fibrosis in rats with bile-duct ligation (BDL).

## Materials and methods

### Animal care

The experimental procedures used in the present study were approved by the Animal Care and Use Committee of Dalian Medical University (Dalian, China), in accordance with the guidelines established by the Canadian Council on Animal Care (Ottawa, ON, Canada). Healthy male Sprague Dawley rats, weighing 200–220 g, were obtained from the laboratory Animal Center of Dalian Medical University.

### Reagents

The following reagents were purchased for the experiments of this study: Rabbit anti-mouse HO-1 antibody (Boster Biological Technology, Wuhan, China), anti-rabbit IgG (MaxVisionTM2; Fuzhou Maixin Biotechnology Development Co., Ltd., Fuzhou, China), Takara RNA polymerase chain reaction (PCR) kit version 3.0 (Avian Myeloblastosis Virus; Takara Bio, Inc., Dalian, China), hydroxyproline (HYP; Nanjing KeyGen Biotech. Co., Ltd., Nanjing, China), cobalt protoporphyrin (CoPP; Sigma-Aldrich, St. Louis, MO, USA) and octreotide (Novartis International AG, Basel, Switzerland).

### Generation of animal models

The rats were divided randomly into four groups: Sham (n=8), cirrhotic (n=8), CoPP treatment (n=12) and octreotide treatment (n=12) groups. The rats were housed in a pathogen-free center that was maintained at room temperature (24–26°C) and 60–65% relative humidity. Water was provided *ad libitum*. The rats were well-fed and housed for three days prior to any experimental procedure. Biliary cirrhosis in the cirrhotic and the octreotide treatment groups was induced by BDL (12). A laparotomy was performed under ether anesthesia. The bile duct was isolated and double-ligated with 3-0 silk. Subsequently, the abdominal wall and the skin were closed with 4-0 silk sutures and antibiotic benzathine benzylpenicillin powder (xxjl Biotechnology Co., Ltd, Wuhan, China) was administered over the closed incision. The rats in the sham group underwent a laparotomy, with the bile duct isolated but not ligated. The rats were continuously fed and housed for a further four-week period following surgery. CoPP was dissolved in 0.2 mol/l NaOH, adjusted to pH 7.4 and diluted in 0.85% NaCl, to obtain a final concentration of 1 mg/ml, as previously described, and was used to induce HO-1 expression (13). The rats in the octreotide treatment and the CoPP groups received an intraperitoneal injection of octreotide (10 μg/kg body weight per day) or CoPP (5 mg/kg body weight per day), respectively, for a week prior to sample collection.

### Sample collection

At four weeks after surgery, the rats were anesthetized with ether and the portal vein and right carotid artery were isolated. A catheter, connected to pressure transducers (BL-420F biological experimental system; Chengdu Technology & Market Co., Ltd., Chengdu, China) was placed in the carotid artery for measurement of mean arterial pressure (MAP), then 1 ml arterial blood was collected in a heparinized syringe through the arterial catheter to measure carboxyhemoglobin (COHb) using a RapidLab 1245 blood gas analyzer (Siemens, Washington, D.C., USA), as an index for the CO level in arterial blood. The catheter was then placed in the portal vein to measure the PVP. Subsequently, 4 ml blood was collected from the rats to measure the serum levels of alanine aminotransferase (ALT), aspartate aminotransferase (AST) (14) and total bilirubin (TBIL) using a Hitachi 7600-110 automatic biochemical analyzer (Hitachi Co., Tokyo, Japan). One lobe of the liver was excised and sections of the tissues were fixed in 10% neutral formalin solution and embedded in paraffin. The remaining tissues were preserved at −80°C for subsequent PCR.

### Hepatic HYP content analysis

The HYP content in the liver, as an indirect index of tissue collagen content, was determined using a Shimadzu UV-1206 spectrophotometer (Shimadzu-Biotech, Kyoto, Japan) and a biological kit (KGT030-2 HYP; Nanjing KeyGen Biotech. Co., Ltd.) according to a modification of a previously described method (15) and is expressed as microgram per gram of wet weight (μg/g).

### Pathological analysis

Hematoxylin and eosin (H&E) and Van Gieson’s (VG) staining were performed according to standard procedures (16). Changes in the liver cells, portal areas and central veins were observed by H&E staining; the proliferation degree of type I collagen was observed with VG staining.

### Reverse-transcription (RT) and qualitative PCR analysis

Total RNA was extracted from the liver by guanidinium phenol-chloroform extraction method. In brief, total RNA was isolated from the liver using TRIzol^®^ reagent (Invitrogen Life Technologies, Carlsbad, CA, USA) according to the manufacturer’s instructions. The homogenate was then centrifuged in order to remove excess proteins, fats and polysaccharides. Following addition of phenol-chloroform (Shanghai BaoMan Biotech. Co., Ltd., shanghai, China), the aqueous phase of the homogenized sample was transferred into a fresh tube. The RNA pellet was precipitated using isopropyl alcohol (Shanghai BaoMan Biotech. Co., Ltd.) through centrifugation (Eppendorf 5417R; 15,000 × g, Eppendorf, Hamburg, Germany). Following washing with 75% ethanol, the dried RNA pellet was dissolved in RNase-free water (Shanghai BaoMan Biotech. Co., Ltd.) and then stored at −70°C. The quantity of the RNA was then determined by measuring the optical density (OD) at 260 nm (A260=1 for 40 ug/ml RNA) and the purity of the RNA was assessed by determining the ratio of the ODs obtained at 260 and 280 nm (pure RNA: A260/A280=2.0) using a Shimadzu UV-1206 spectrophotometer. The primer sequences were as follows: HO-1 forward: 5′-ACT TTC AGA AGG GTC AGG TGT CC-3′ and reverse: 5′-TTG AGC AGG AAG GCG GTC TTA G-3′ (product size, 524 bp); β-actin forward: 5′-GGA GTC AAC GGA TTT GGT-3′ and reverse: 5′-GTG ATG GGA TTT CCA TTG-3′ (product size, 226 bp). An aliquot of each RT reaction mixture was used for PCR amplification and the PCR products were separated by 2.5% agarose gel electrophoresis. Images of the product bands were captured and the density of each product band was quantified and expressed as the ratio of the band density for HO-1 mRNA to that of β-actin mRNA.

### Western blotting

A volume of 1 ml lysate, containing 20 mmol/l Tris (pH 7.5), 150 mmol/l NaCl, 1% Triton X-100 and 1 mmol/l phenylmethanesulfonylfluoride, was added to 100 mg liver tissue. The mixture was homogenized by centrifugation at 3,500 × g for 3–5 min at 4°C and the supernatant was separated and quantified, as previously described (17). Following SDS-PAGE, the sample was transferred to a polyvinylidine fluoride membrane and stained with 3,3′-diaminobenzidine (Maixin Biotechnology Co., Ltd, Fujian, China). The sample was incubated with primary (rabbit-anti-mouse HO-1 monoclonal antibody, 1:100) and secondary antibody (peroxidase-labeled sheep-anti-rabbit antibody, 1:100), with β-actin serving as an internal reference.

### Immunohistochemical analysis

The liver tissues were fixed in a 10% neutral formalin solution, embedded in paraffin wax and cut into sections. A proportion of the sections were stained with H&E, while the other sections underwent deparaffinization, rehydration and inactivation, and were then incubated with rabbit-anti-mouse HO-1 monoclonal antibody (1:50) at room temperature for 60 min, followed by incubation with secondary antibody (1:1,500; MaxVisionTM2 kit; Maixin Biotechnology, Fuzhou, China) at room temperature for 15 min. The sections were mounted subsequent to staining. Primary antibody was replaced by phosphate-buffered saline for use as a negative control. Images were captured and analyzed using Image-Pro-Plus 6.0 software (Media Cybernetics, Rockville, MD, USA) to calculate the area and mean density of positive expression. The from five visual fields were averaged from each sample.

### Statistical analysis

Data analysis was performed using SPSS 10.0 software (SPSS, Inc., Chicago, IL, USA). Analysis of variance and Wilcoxon statistical methods were used to determine statistical significance. All measurements are expressed as the means ± standard deviation. P<0.05 was considered to indicate a statistically significant difference.

## Results

### Biochemical examination

At four weeks after surgery, bile duct dilation was observed in the BDL group, and ascites and jaundice had also developed. This indicated that the cirrhotic model was successfully induced by BDL.

The serum levels of AST, ALT and TBIL in the cirrhotic group were significantly higher than those in the sham group [313.63±10.65 versus 166.6±7.27 IU/l (P<0.01), 51.25±4.05 versus 38.13±3.20 IU/l (P<0.05) and 10.54±0.22 versus 0.83±0.23 μmol/l (P<0.01), respectively]. As compared with the cirrhotic group, the serum levels of AST, ALT and TBIL were significantly lower in the octreotide-treated group (205.08±8.03 versus 313.63±10.65 IU/l, 42.37±2.59 versus 51.25±4.05 IU/l and 6.85±0.21 versus 10.54±0.22 μmol/l, respectively; P<0.05) and significantly higher in the CoPP group [406.66±14.79 versus 313.63±10.65 IU/l (P<0.05), 72.04±3.52 versus 51.25±4.05 IU/l (P<0.05) and 12.02±0.47 versus 10.54±0.22 umol/l (P<0.01; Table I].

### Hemodynamic parameters and arterial blood gas levels

The PVP was significantly higher and the MAP was significantly lower in the cirrhotic group, as compared with those in the sham group [15.54±2.32 versus 9.13±0.62 cm H_2_O (P<0.05), 59.28±12.17 versus 116.06±7.31 mmHg (P<0.01), respectively]. As compared with the cirrhotic group, the PVP was significantly higher in the CoPP group (17.38±1.20 versus 15.54±2.32 cmH_2_O; P<0.05). The octreotide-treated rats exhibited significantly reduced PVP, as compared with the BDL cirrhotic rats (13.12±1.10 versus 15.54±2.32 cm H_2_O, P<0.05), but did not exhibit significantly different MAP values (65.20±4.52 versus 59.28±12.17 mm Hg; P>0.05). The levels of COHb in the arterial blood were significantly higher in the cirrhotic group as compared with those in the sham group (0.50±0.20 versus 0.23±0.05%; P<0.01). As compared with the cirrhotic group, the octreotide treatment group exhibited significantly reduced COHb levels (0.24±0.07 versus 0.50±0.20%; P<0.01), while the CoPP treatment group exhibited significantly increased COHb levels (0.83±0.39 versus 0.50±0.20%; P<0.05; Table II).

### Liver histopathological analysis in each experimental group of rats

**Figure 1:**
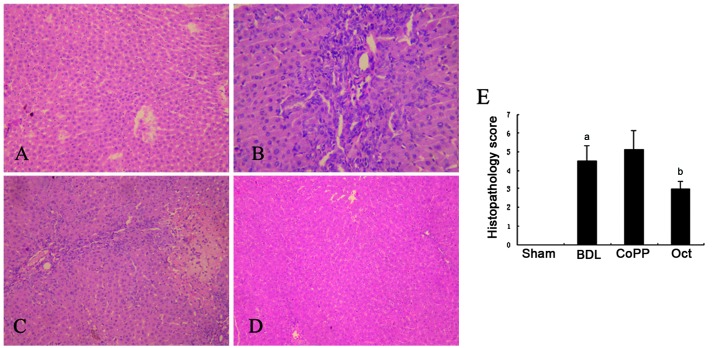
Representative photomicrographs showing pathological changes in the liver using hematoxylin and eosin staining. Magnification, ×100. (A) Liver structure in the Sham group; (B) liver structure in the bile duct ligation (BDL) cirrhosis group; (C) liver structure in the octreotide treated group (Oct) and (D) liver structure in the cobalt protoporphyrin (CoPP) group. (E) Histopathological fibrosis scores. Values are expressed as the mean ± standard error of the mean. ^a^P<0.01 vs. sham group; ^b^P<0.05 vs. BDL group.

Liver tissue samples from rats of each experimental group were stained by H&E and VG to examine the histopathological changes. In the sham group, structural integrity of the hepatic lobule was observed with no proliferation of fibrous tissue. Only a few small collagen fibers were visible in the portal areas (Fig. 1A). In the cirrhotic rats four weeks after BDL, significant proliferation of fibrous tissue in the portal areas, widened lobular septa with greater fiber deposition, infiltration of numerous inflammatory cells in the portal area and the area surrounding the central vein, and formation of fibrous septa were observed (Fig. 1B). As compared with the cirrhotic group, proliferation of fibrous tissue was more marked in the CoPP group (Fig. 1C). In the octreotide treatment group, fibrous hyperplasia was significantly reduced, fine fibers were occasionally observed, being distributed mainly in the portal areas, and a few inflammatory cells were detected in the portal area and surrounding area of the central vein (Fig. 1D).

**Figure 2:**
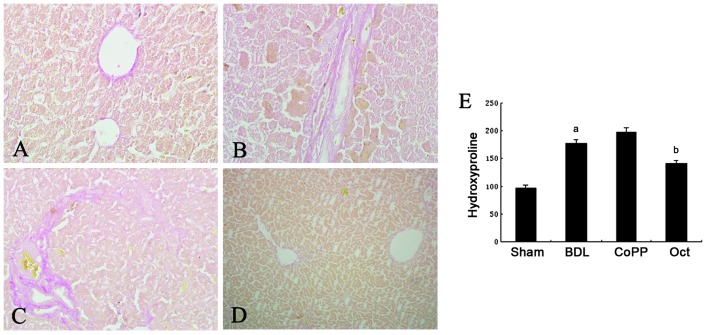
Van Gieston’s staining of collagen type I in liver sections and the liver hydroxyproline content. Magnification, ×100. (A) Collagen type I was marginally detected in the sham group; Collagen type I was deposited to a greater extent in (B) the bile duct ligation group, (D) the cobalt protoporphyrin (CoPP) group and (C) less deposited in the octreotide-treated group (Oct). (E) Liver tissue hydroxyproline content in the different groups. The values are expressed as the means ± standard error of the mean. ^a^P<0.01 vs. sham group; ^b^P<0.05 vs. bile duct ligation (BDL) group.

The presence of collagen type I in the liver tissue was observed by VG staining. In the BDL group (Fig. 2B), the collagen type I in the portal area and bile duct wall was markedly thicker as compared with the sham group (Fig. 2A; P<0.01). As compared with the BDL group, the extent of fibrosis was markedly higher in the CoPP group (Fig. 2C) and was significantly lower in the octreotide treatment group (Fig. 2D; P<0.05).

The changes in the HYP content in the liver tissue between the different groups (Fig. 2E) were in accordance with those of type I collagen. As compared with the sham group, the HYP content was significantly higher in the BDL and CoPP groups (P<0.01). As compared with the BDL group, the HYP content was significantly lower in the octreotide treatment group (P<0.05).

### Immunohistochemical detection of hepatic HO-1 protein in each experimental group of rats

**Figure 3:**
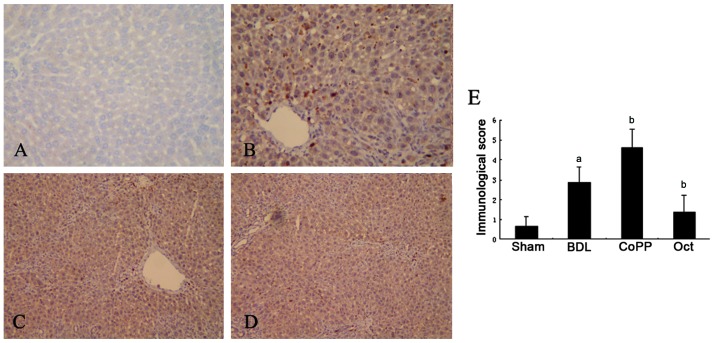
Hepatic heme oxygenase-1 (HO-1) protein expression. Immunohistochemical staining of hepatic HO-1 protein expression in rats in (A) the sham group, (B) the cirrhotic group, (C) the octreotide-treated group (Oct) and (D) the cobalt protoporphyrin (CoPP) group. Magnification, ×100. (E) Scores of immunohistochemical staining of hepatic HO-1 protein expression in each group. ^a^P<0.01 vs. sham group; ^b^P<0.05 vs. bile duct ligation (BDL) group.

To localize HO-1 protein expression in the liver samples, immunohistochemistry was performed using specimens from the four groups. As shown in Fig. 3, the expression of HO-1 protein was mainly located in Kupffer’s cells and hepatocytes, similar to previously reported findings (18,19). The intensity and percentage of cells expressing HO-1 protein in the liver were also analyzed. Mild staining was observed in hepatic tissue samples from the sham group, with a score of 0.63±0.52. HO-1 immunoreactivity was strongly positive in the cirrhotic group, with a score of 4.17±0.58, which was significantly higher than that of the sham group, (P<0.01). Octreotide treatment significantly reduced the HO-1 immunostaining in the cirrhotic rats with a score of 1.21±0.36 (P<0.01; Fig. 3).

### Hepatic HO-1 protein expression levels detected by western blot analysis in each experimental group of rats

**Figure 4:**
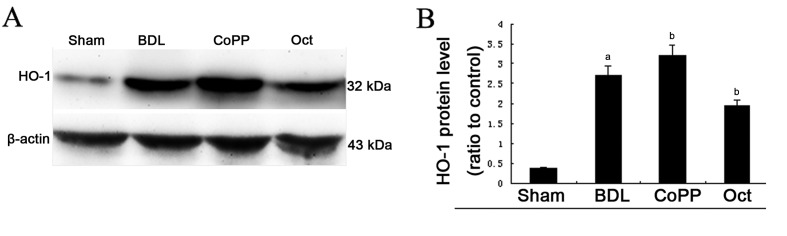
Hepatic heme oxygenase-1 (HO-1) protein expression levels, detected by western blot analysis. (A) Hepatic HO-1 protein expression levels were greater in the bile duct ligation (BDL) group than in the Sham group; the levels were significantly higher in the cobalt protoporphyrin (CoPP) group and lower in the octreotide group (Oct), compared with the cirrhotic group. (B) Quantitative data: The ratio of the corresponding hepatic HO-1 protein expression levels to those of β-actin. ^a^P<0.01 vs. sham group; ^b^P<0.05 vs. BDL group. kDa, kilodaltons.

Western blot analysis revealed that the protein expression levels of HO-1 were significantly higher in the BDL group compared with those of the sham group (P<0.01; Fig. 4); in addition, HO-1 expression was increased in the CoPP group and decreased in the octreotide group compared with that of the BDL group.

### Hepatic HO-1 mRNA expression levels in each experimental group of rats

**Figure 5:**
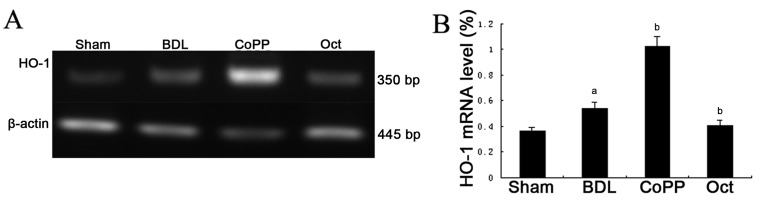
Hepatic heme oxygenase-1 (HO-1) mRNA expression levels. (A) Representative reverse transcription polymerase chain reaction data revealed that the HO-1 mRNA expression levels were higher in the cirrhotic group than in the Sham group; the levels were significantly higher in the cobalt protoporphyrin (CoPP) group and significantly lower in the octreotide group (Oct) than in the cirrhotic group. (B) Quantitative data of the ratio of corresponding hepatic HO-1 mRNA band density to that of β-actin mRNA. ^a^P<0.01 vs. sham group; ^b^P<0.05 vs. BDL group.

As determined by reverse transcription PCR, the hepatic expression levels of HO-1 mRNA in the cirrhotic group were significantly higher than those in the sham group (P<0.01). The hepatic HO-1 mRNA expression levels were significantly reduced in the octreotide-treated group, as compared with those in the cirrhotic group. (P<0.05; Fig. 5).

## Discussion

Liver cirrhosis is a chronic scarring process in the liver and is the predominant cause of portal hypertension. The most direct consequence of portal hypertension is the development of gastroesophageal varices that may rupture and result in the development of variceal hemorrhage (20,21). Octreotide has been widely used in the clinical practice and has shown significant efficacy in the control of oesophageal variceal hemorrhage (3). Octreotide is a synthetic 8-amino-acid analogue of somatostatin with a prolonged action. Octreotide shares four amino acids with somatostatin and exerts similar biological effects to the native hormone (22). Octreotide binds to somatostatin receptors, which are coupled via pertussis toxin-sensitive G proteins. This results in contraction of the splanchnic arteries through inhibition of adenylyl cyclase (23). The effect of contraction of the splanchnic arteries results in a decrease in the functional portal blood flow. Octreotide has been shown to significantly reduce portal pressure in patients with portal hypertension, with marginal effects on systemic hemodynamics (24,25).

Previous studies have shown that the HO-1 is overexpressed in patients and models with liver cirrhosis and contributes to portal hypertension (10,11). The aim of the present study was to investigate whether octreotide regulates hepatic HO-1 expression while reducing PVP, and the influence of altered hepatic HO-1 expression levels on hepatic function and fibrosis in liver cirrhotic rats.

The results of the present study demonstrated that the serum levels of ALT, AST and TBIL were significantly higher in the cirrhotic group as compared with those in the sham group, indicating that BDL results in marked liver injury, with liver cirrhosis confirmed by H&E staining in the livers of the BDL rats. In addition, the MAP was significantly lower in the BDL rats as compared with the time-matched sham rats, indicating that a hyperdynamic state had occurred. Furthermore, the PVP was significantly higher in the BDL rats as compared with the time-matched sham rats, indicating a condition of portal hypertension. The results suggested that a BDL model was successfully established in the experiments. In addition, the levels of COHb were significantly higher in the biliary cirrhosis group than those in the sham group, suggesting that overproduction of CO occurred in the cirrhotic rats, since CO is found predominately bound to hemoglobin in the form of COHb in the circulation (26). HO-1 is the primary source of circulating CO (27) and contributes to vasodilation mainly through HO-1-derived CO (28). Hepatic HO-1 mRNA and protein expression levels were also found in the present study to be significantly increased in the cirrhotic group, as compared with the sham group, which is consistent with the results of a previous study (10).

The results of the present study revealed that octreotide treatment significantly reduced the production of CO in arterial blood and the HO-1 expression levels, as compared with the BDL-only group. This was accompanied by a reduction in PVP in the octreotide rats, compared with the cirrhotic rats, but no significant differences in MAP values were detected between these two groups. CO, a gaseous messenger similar to NO, activates soluble guanylate cyclase, resulting in the generation of cyclic guanosine monophosphate (cGMP) (29), which then mediates various physiological functions, including vasodilation (30). CO relaxes vascular smooth muscle by a cGMP-independent mechanism (31,32). The CO-mediated vasodilation is not systemic, but is mainly localized on certain vascular beds, such as the splanchnic vascular bed (33). Splanchnic arterial vasodilation results in a reduced vascular resistance, which increases portal venous inflow and maintains the elevated PVP (34). Thus, reduction of the HO-1/CO system, resulting from octreotide administration in portal hypertensive rats, may involve octreotide-induced decreased functional portal blood flow. Since CO-mediated vasodilatation is mainly localized on splanchnic vascular beds (33), this may explain why the octreotide treatment did not significantly affect the MAP in cirrhotic rats, although it reduced the portal pressure.

CoPP was used for inducing HO-1. The present study demonstrated that overproduced CO, from increased HO-1, aggravated portal hypertension in the CoPP group. This effect was accompanied by increased HO-1 expression levels. The serum levels of ALT, AST and TBIL were significantly higher in the CoPP group than those in the cirrhotic group, and the extent of fibrosis was markedly higher in the CoPP group than that in the cirrhotic group. However, inhibition of HO-1 by octreotide administration significantly reduced the serum levels of AST, ALT and TBIL in the cirrhotic rats, and attenuated liver fibrosis. Previous studies have also indicated that overexpression of HO-1 is harmful to liver function and aggravates liver fibrosis in BDL rats (35–37). Although certain studies have observed that HO-1 is protective in liver cells in various liver diseases (38–41), and upregulation of HO-1 interfered with chronic inflammation and prevented progression of liver fibrosis in Mdr2-knockout mice (42), the present study demonstrated the opposite result. The reasons for these differences results may be that HO-1 exerts different roles during the progression of liver fibrosis (43) and that protection is restricted to a narrow HO-1 expression threshold (44). In the early stages of liver fibrosis, low HO-1 induction may exert a protective action (45), but in the end stages of cirrhosis with portal hypertension, excessive HO-1 expression deteriorates liver function and aggravates liver cirrhosis (43,46,47).

In conclusion, the present study demonstrated that administration of octreotide inhibited hepatic HO-1 overexpression in cirrhotic rats, reduced hepatic HO-1 expression levels, relieved liver injury and attenuated liver fibrosis.

## Figures and Tables

**Table I:** Comparison of serum ALT, AST and TBIL in the different groups.

Group	ALT (IU/l)	AST (IU/l)	TBIL (μmol/l)
Sham group	38.13±3.20	166.61±7.27	0.83±0.23
Cirrhotic group	51.25±4.05^b^	313.63±10.65^a^	10.54±0.22^a^
CoPP group	72.04±3.52^d^	406.66±14.79^d^	12.02±0.47^c^
Octreotide group	42.37±2.59^d^	205.08±8.03^d^	6.85±0.21^d^

a P<0.01,

b P<0.05 vs. Sham group;

c P<0.01,

d P<0.05 vs. Cirrhotic group.

ALT, alanine aminotransferase; AST, aspartate aminotransferase; TBIL, total bilirubin; CoPP, cobalt protoporphyrin.

**Table II:** Comparison of PVP, MAP and COHb in the different groups.

Group	PVP (cmH_2_O)	MAP (mmHg)	COHb (%)
Sham group	9.13±0.62	116.06±7.31	0.23±0.05
Cirrhotic group	15.54±2.32^b^	59.28±12.17^a^	0.50±0.20^a^
CoPP group	17.38±1.20^d^	52.79±5.74	0.83±0.39^d^
Octreotide group	13.12±1.10^d^	65.20±4.52	0.24±0.07^c^

a P<0.01,

b P<0.05 vs. Sham group;

c P<0.01,

d P<0.05 vs. Cirrhotic group.

PVP, portal vein pressure; MAP, mean arterial pressure; COHb, carboxyhemoglobin; CoPP, cobalt protoporphyrin.
